# Social media communication and digital learning inclusion: effects on student wellbeing and social change in higher education

**DOI:** 10.3389/fpsyg.2026.1910919

**Published:** 2026-08-11

**Authors:** Nimet Harmanci

**Affiliations:** Faculty of Business, Department of Business Administration, University of Mediterranean Karpasia, North Cyprus, Türkiye

**Keywords:** digital learning inclusion, higher education equity, social change orientation, social media communication, student wellbeing

## Abstract

While social media has become integral to higher education, limited research examines its mechanisms for improving student wellbeing and social consciousness. This study addresses the gap by investigating whether digital learning inclusion mediates the relationship between social media communication and student outcomes, and how this pathway varies by institutional and individual factors. A cross-sectional survey of 650 students from multiple universities employed Partial Least Squares Structural Equation Modeling (PLS-SEM) to test direct and indirect effects, complemented by multi-group and moderation analyses. Results reveal that an estimated 54% of social media communication’s association with wellbeing, and 24% of its association with social change orientation, operated indirectly through digital learning inclusion, with substantial moderation by digital literacy (38.9% effect difference), institutional support (37.5%), and socioeconomic status. These findings extend Social Cognitive Theory and Digital Inclusion frameworks by foregrounding mechanisms over technologies and demonstrating that institutional equity commitments were associated with more effective use of social media for holistic student development. The study offers evidence-based guidance for institutional practice and identifies equity disparities requiring targeted intervention.

## 1 Introduction

Higher education institutions face unprecedented challenges and opportunities in the digital age. The rapid proliferation of social media platforms and digital learning technologies has fundamentally transformed how students engage with academic content, interact with peers, and participate in educational communities (Grieve et al., 2013). Substantial empirical evidence supports the premise that social media communication is associated with educational outcomes (Ironsi, 2023a). Junco and Cole-Avent (2018) found that student engagement on Twitter for course-related purposes correlated significantly with improved academic outcomes and greater sense of community. Similarly, research by Wang et al. (2012) demonstrated that social media platforms facilitate peer interaction and collaborative learning, creating opportunities for knowledge co-construction that traditional classroom settings alone may not provide.

The connection between digital communication and student wellbeing presents a more complex picture in the literature. While some studies document positive associations between social media use and social connectedness (Grieve et al., 2013), others reveal that excessive social media engagement correlates with anxiety, depression, and reduced academic performance (Throuvala et al., 2021).

This apparent contradiction is resolved when considering contextual factors: the quality of social media interactions, the degree to which digital communication supports academic goals, and institutional provisions for digital inclusion significantly mediate the relationship between social media use and wellbeing. Recent meta-analyses (Orben and Przybylski, 2019) suggest that the relationship between social media and psychological outcomes is modest and conditional on how students utilize these platforms.

Digital learning inclusion has emerged as a critical educational construct in response to persistent inequities in technology access and capability (Selwyn, 2019; Ironsi, 2022). Research demonstrates that merely providing technology does not ensure inclusive learning outcomes (Ironsi, 2023a). Rather, inclusion requires deliberate design of learning environments that consider diverse student needs, ensure accessibility, promote equitable participation, and build digital competencies (Bates, 2015). Institutions implementing comprehensive digital inclusion frameworks report enhanced student engagement and satisfaction (Martin and Bolliger, 2018). However, few studies examine how digital learning inclusion mediates the relationship between social media communication and student outcomes, representing a significant gap in the literature.

Social change orientation—encompassing civic engagement, digital activism, and commitment to social justice—has long been considered an important outcome of higher education (Dewey, 1916; Boyes-Watson and Pranis, 2015). Contemporary research suggests that digital platforms provide unprecedented opportunities for students to engage in activism and social advocacy (Rotman and Preece, 2010; Bennett, 2008).

However, the mechanisms through which social media communication fosters social change orientation remain incompletely understood. Some research suggests that online activism may substitute for, rather than complement, face-to-face civic engagement (the “slacktivism” debate), while other evidence indicates that digital platforms effectively mobilize collective action and social awareness, particularly among younger populations (Boulianne, 2015). The role of institutional support and digital literacy in shaping the relationship between social media use and social change orientation requires empirical investigation.

Despite substantial research on social media in education and digital learning technologies, several critical gaps persist in the literature. First, relatively few studies examine digital learning inclusion as a mediating mechanism linking social media communication to student outcomes. While researchers have investigated how digital inclusion affects learning outcomes (Bates, 2015), and separately examined social media’s association with student engagement (Junco and Cole-Avent, 2018), the integrated pathway remains underspecified. This gap is particularly significant because institutional design of digital learning environments may determine whether social media communication yields benefits or harms for student wellbeing.

Second, the literature provides limited understanding of how social media communication is associated with social change orientation among higher education students. While studies document the role of online platforms in activism (Rotman and Preece, 2010), few examine the mechanisms through which educational social media use translates into sustained social change orientation. This gap is consequential because higher education carries a historic commitment to developing engaged citizens and agents of social change (Boyes-Watson and Pranis, 2015). Understanding these pathways would inform institutional efforts to leverage digital technologies for civic learning.

Third, research has insufficiently examined moderating factors that determine when and for whom social media communication produces beneficial outcomes. While studies acknowledge that digital literacy shapes technology use (Van Deursen and Van Dijk, 2019), few empirically test moderation effects across multiple individual and institutional factors simultaneously. Recent scholarship emphasizes AI readiness as an emerging competency (Ng et al., 2022), yet its moderating role in the relationship between social media communication and digital learning inclusion has not been empirically examined. Similarly, socioeconomic status and gender remain understudied as moderators of technology-education relationships in higher education contexts, despite documented disparities in access and outcomes (Selwyn, 2019).

Addressing these research gaps bears significant implications for multiple stakeholder groups. For institutional leaders, understanding the relationships between social media communication, digital learning inclusion, and student outcomes provides evidence for resource allocation and policy decisions regarding technology investment and digital learning design. The COVID-19 pandemic accelerated reliance on digital learning technologies and social media for academic communication, intensifying the urgency of this research (Dhawan, 2020). Many institutions will continue hybrid or online delivery models indefinitely, making it critical to understand how to optimize digital learning environments for student wellbeing.

This research employs a multi-stage analytical strategy combining Partial Least Squares Structural Equation Modeling (PLS-SEM) to address these issues. This methodological approach goes beyond identifying net effects to reveal configurations of conditions that is associated with optimal student outcomes—insights particularly valuable for higher education practitioners seeking to optimize digital learning environments. The dual-method framework addresses calls in the literature for more nuanced analytical approaches that capture the complexity of educational phenomena (Rihoux and Ragin, 2009).

The present study makes several distinct contributions to the literature. First, it empirically validates an integrated model linking social media communication to student wellbeing through digital learning inclusion as a mediating mechanism, addressing a theoretical and empirical gap in the educational technology literature. Second, it extends understanding of social media’s role in developing social change orientation, connecting educational communication research to civic engagement literature. Third, it examines moderating effects of digital literacy, gender, socioeconomic status, and institutional support, and additionally profiles online learning self-efficacy and AI readiness as descriptive individual-difference measures within the sample, recognizing that outcomes vary across student populations and institutional contexts. These contributions collectively advance theoretical understanding while providing actionable evidence for institutional practice.

The research questions guiding this investigation are:

- RQ1: How does social media communication influence digital learning inclusion among higher education students?
- RQ2: Does digital learning inclusion improve student wellbeing?
- RQ3: What role does social media communication play in shaping students’ social change orientation?
- RQ4: Does digital learning inclusion mediate the relationship between social media communication and student wellbeing?
- RQ5: To what extent do digital literacy, institutional support, socioeconomic status, and gender moderate the relationships among social media communication, digital learning inclusion, student wellbeing, and social change orientation? (Online learning self-efficacy and AI readiness were assessed descriptively and examined at the correlational level; see sections 4.3 and 5.1.)

To address these questions, the study tests the following hypotheses.

H1 proposes that social media communication is positively associated with digital learning inclusion.

H2 posits that digital learning inclusion is positively associated with student wellbeing. H3 suggests that social media communication is positively associated with social change orientation.

H4 hypothesizes that digital learning inclusion mediates the relationship between social media communication and student wellbeing. H5 and H6 propose moderating effects of digital literacy and institutional support, respectively, on key pathways. These hypotheses translate the research questions into testable propositions that guide the empirical investigation. The remainder of this article is organized as follows. Section 2 provides comprehensive literature review addressing social media communication in educational contexts, digital learning inclusion as an equity and access issue, theoretical frameworks explaining student wellbeing, and social change orientation as an educational outcome. Section 3 presents the methodology, including research design, sampling strategy, measurement instruments, data collection procedures, and ethical considerations. Section 4 reports results from preliminary analyses, measurement model validation, structural model testing, and sophisticated extensions including multi-group analysis and findings. Section 5 discusses theoretical implications, practical applications, and comparison with prior research. Section 6 concludes with policy recommendations, limitations, and directions for future research.

## 2 Literature review and theoretical framework

### 2.1 Social media communication in higher education

Social media has become an integral component of higher education, transforming communication, collaboration, and learning (Junco, 2012; Barczyk and Duncan, 2013). Platforms such as Facebook, Twitter, Instagram, LinkedIn, and Discord are increasingly used to facilitate student engagement, academic interaction, and flexible learning opportunities (Greenhow and Lewin, 2016; Manca and Ranieri, 2016). When purposefully integrated into teaching practices, social media has been associated with improved academic performance, stronger interaction between students and instructors, and an enhanced sense of belonging.

These benefits are largely explained by the concept of social presence, which refers to students’ perceptions of connectedness within digital learning environments (Lowenthal and Dunlap, 2014; Richardson and Lowenthal, 2017). Structured interactions through social media strengthen social presence and student engagement (Wang et al., 2012), although the quality of interaction is critical. Meaningful dialogue, instructor facilitation, and alignment with course assessments distinguish effective educational use from superficial implementation (Martin and Bolliger, 2018).

Nevertheless, empirical findings remain mixed. While several studies report positive associations between academic social media use and learning outcomes, others find little advantage over traditional instructional approaches. Collectively, this evidence suggests that social media contributes to learning only when intentionally embedded within pedagogical activities rather than adopted as a technological add-on.

Although previous studies have established the educational potential of social media communication, limited research has examined how these communication practices contribute to digital learning inclusion and subsequent student wellbeing and social change within a single integrated framework, thereby motivating the present study.

### 2.2 Digital learning inclusion: equity, access, and participation

Digital learning inclusion extends beyond access to technology and encompasses equitable participation, accessibility, and the removal of barriers to learning (Selwyn, 2019). The COVID-19 pandemic exposed persistent inequalities in internet connectivity and device availability, disproportionately affecting students from lower-income households and reinforcing existing educational disparities (Dhawan, 2020; Zuboff-Rose, 2021).

Research consistently demonstrates that access to reliable internet services and appropriate digital devices varies across socioeconomic groups, influencing students’ capacity to participate effectively in online learning (Zhou et al., 2025). However, access alone does not ensure inclusion. Accessibility features, digital literacy, and institutional support collectively determine the quality of students’ digital learning experiences (Bates, 2015; Mexhuani, 2025). Accessible technologies such as captioning and screen readers, together with digital skills training and institutional investment in technological infrastructure, are essential for equitable educational outcomes (Van Deursen and Van Dijk, 2019; Martin and Bolliger, 2018).

Digital literacy further shapes students’ ability to participate effectively in technology-mediated learning (Zuo et al., 2026). It encompasses technical, informational, communicative, and critical competencies required for successful technology use (Zuo et al., 2026). Students enter higher education with varying levels of digital literacy influenced by socioeconomic background, previous technological exposure, and educational opportunities (Ironsi, 2023b). Those with limited digital competencies often encounter difficulties navigating learning platforms and accessing educational resources. Consequently, digital learning inclusion should be understood as a multidimensional construct comprising access, accessibility, digital literacy, and institutional support rather than technology provision alone.

Despite growing recognition of digital learning inclusion as a multidimensional construct, relatively little empirical research has examined its mediating role in linking social media communication with student wellbeing and broader social change outcomes, providing an important gap addressed by this study.

### 2.3 Student wellbeing in digital learning environments

Student wellbeing has become a major concern in higher education because of increasing levels of anxiety, depression, loneliness, and academic stress (American College Health Association, 2021; Lipson et al., 2019). Within digital learning environments, wellbeing is shaped not only by access to technology but also by the nature of students’ online interactions (Gelles et al., 2020).

Evidence suggests that social media can strengthen social connectedness and belonging by enabling students to maintain relationships, participate in supportive communities, and engage in academic collaboration (Grieve et al., 2013; Baumeister and Leary, 1995). Students who actively use social media for communication and collaborative learning frequently report stronger feelings of belonging and community.

However, research also demonstrates that the role of social media depend on how it is used. Passive or excessive engagement is associated with anxiety, depression, and negative social comparison (Vogel et al., 2014; Orben and Przybylski, 2019). In contrast, active engagement—including communication, collaboration, and community participation—has consistently been linked to more positive wellbeing outcomes (Yap and Lim, 2024; Throuvala et al., 2021). This distinction between active and passive engagement suggests that opportunities for meaningful interaction are central to promoting psychological wellbeing within digitally mediated learning environments.

Although prior research has distinguished between beneficial and harmful patterns of social media use, insufficient attention has been given to the mechanisms through which digital learning inclusion may enhance the positive effects of academic social media communication on student wellbeing, thereby justifying the proposed mediation model.

### 2.4 Social change orientation and digital activism

Higher education has long sought to cultivate socially responsible and civically engaged citizens (Dewey, 1916). Social media has expanded opportunities for civic participation by facilitating awareness creation, advocacy, public dialogue, and collective action (Bennett, 2008; Boulianne, 2015; Rotman and Preece, 2010).

Despite these opportunities, concerns regarding “slacktivism” suggest that online participation does not always translate into sustained civic engagement or meaningful social action (Boulianne, 2015). Research indicates that universities can strengthen students’ social change orientation by embedding social justice education, service learning, and community engagement within academic curricula (Einfeld and Collins, 2008). Institutional support therefore plays a critical role in transforming digital engagement into meaningful civic participation by connecting online interaction with real-world action.

Taken together, the evidence indicates that social media possesses considerable potential to foster social change orientation, but its effectiveness depends on purposeful educational design and institutional guidance that encourage meaningful participation beyond online interaction alone.

Existing studies have largely examined digital activism or civic engagement independently; however, limited research has investigated how social media communication and digital learning inclusion jointly contribute to students’ social change orientation within a comprehensive higher education model, which constitutes the final research gap addressed in this study.

### 2.5 Theoretical frameworks

This study draws upon three complementary theoretical perspectives: Social Cognitive Theory (Bandura, 1986), Uses and Gratifications Theory (Katz, 1959; Ruggiero, 2000), and Digital Inclusion Theory (Selwyn, 2019).

#### 2.5.1 Social cognitive theory

Social Cognitive Theory (Bandura, 1986) explains human behavior as the result of reciprocal interactions among personal, behavioral, and environmental factors. Within digital learning environments, the theory highlights the importance of self-efficacy, observational learning, and environmental affordances in shaping students’ engagement with social media and educational technologies (Artino, 2012).

#### 2.5.2 Uses and gratifications theory

Uses and Gratifications Theory (Katz, 1959; Ruggiero, 2000) explains media use as an active process driven by individuals’ needs and motivations. Students engage with social media for information seeking, academic collaboration, social interaction, and entertainment; the gratifications obtained shape both the frequency and quality of their engagement with digital learning environments (Junco, 2012; Junco and Cole-Avent, 2018).

#### 2.5.3 Digital inclusion theory

Digital Inclusion Theory focuses on equity within technology-enhanced education, arguing that meaningful inclusion requires access to technology, opportunities for participation, and equitable outcomes (Selwyn, 2019; Van Deursen and Van Dijk, 2019; Ironsi, 2026).

The theory highlights that technology provision alone is insufficient: students facing socioeconomic disadvantages, accessibility barriers, or limited digital skills may remain excluded despite access to digital tools (Mexhuani, 2025; Zuboff-Rose, 2021). Institutions must therefore address structural barriers to ensure full participation (Dhawan, 2020) excluded despite having access to digital tools. Therefore, institutions must address structural inequalities through inclusive policies and support services.

### 2.6 Moderating factors

Technology outcomes vary by individual and contextual characteristics (Van Deursen and Van Dijk, 2019). Digital literacy is a primary moderator: students with stronger competencies achieve better academic and psychological outcomes (Koehler et al., 2015). Socioeconomic status, gender, online learning self-efficacy, and AI readiness also shape the benefits students derive from digital learning environments (Ng et al., 2022), underscoring the importance of testing moderation effects empirically.

### 2.7 Research gaps and study rationale

Key gaps include: (1) limited examination of digital learning inclusion as a mediating mechanism; (2) insufficient explanation of how social media fosters sustained social change orientation; (3) rarely tested moderation effects of digital literacy, SES, and institutional support simultaneously; and (4) reliance on symmetric methods that overlook configurational complexity.

#### 2.7.1 Integrated conceptual framework

**Figure 1:**
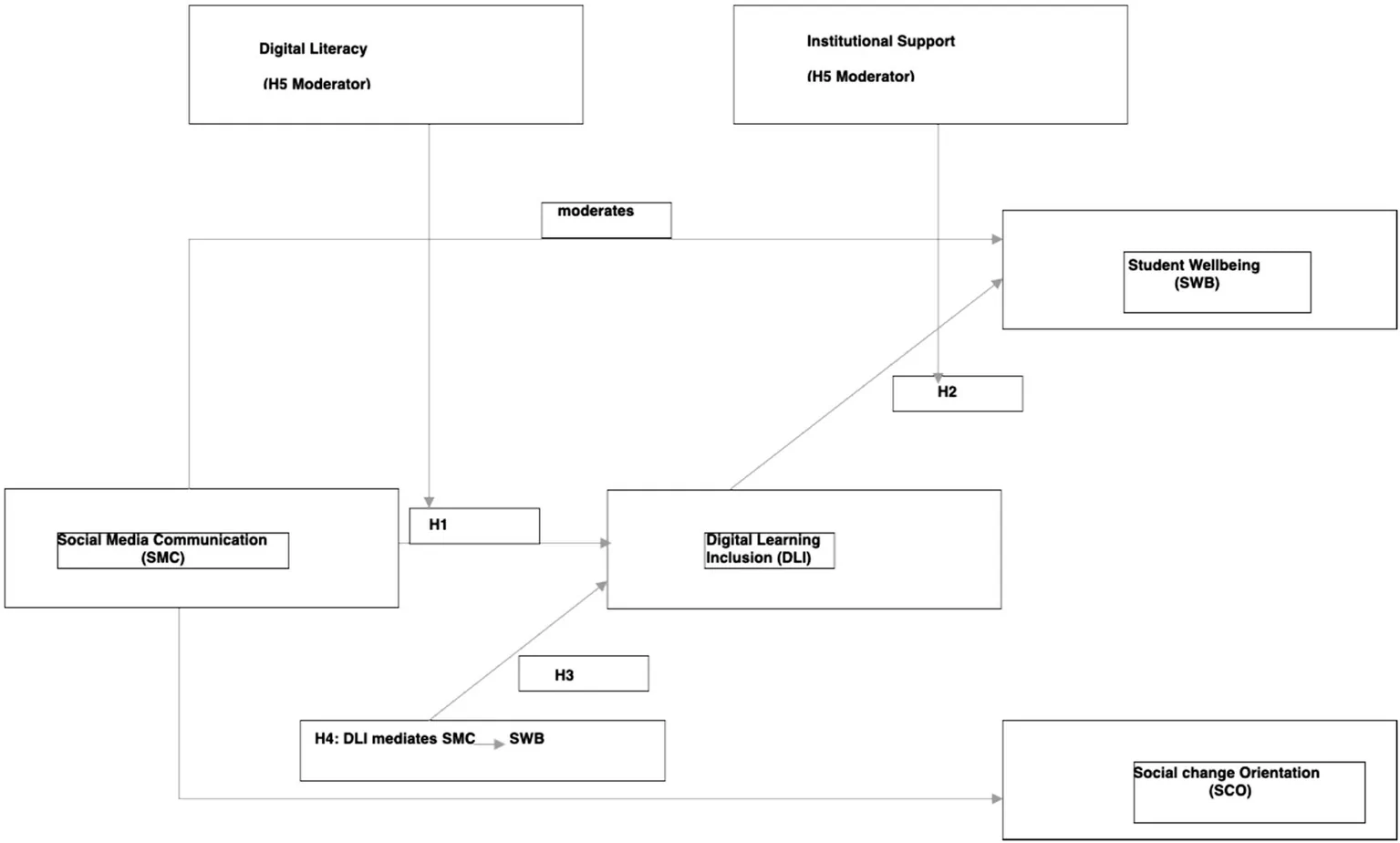
Conceptual framework model.

This study integrates Social Cognitive Theory (Bandura, 1986), Uses and Gratifications Theory (Katz, 1959; Ruggiero, 2000), and Digital Inclusion Theory (Selwyn, 2019; Van Deursen and Van Dijk, 2019) into a unified framework. SCT explains how self-efficacy and institutional environments shape engagement; UGT explains why students use social media for informational, social, and educational needs (Junco and Cole-Avent, 2018); and Digital Inclusion Theory situates inequities in access, participation, and outcomes. Together, these frameworks predict that digital literacy and institutional support amplify the positive effects of social media communication on inclusion and student wellbeing. This conceptual framework is presented in Figure 1.

The model generates six primary hypotheses structured around direct, mediated, and moderated pathways:

*H1*: Social media communication is positively associated with digital learning inclusion.

*H2*: Digital learning inclusion is positively associated with student wellbeing.

*H3*: Social media communication is positively associated with social change orientation.

*H4*: Digital learning inclusion mediates the relationship between social media communication and student wellbeing.

*H5*: Digital literacy positively moderates the effect of social media communication on digital learning inclusion, such that the effect is stronger for students with higher digital literacy.

*H6*: Institutional support positively moderates the effect of digital learning inclusion on student wellbeing, such that the effect is stronger in contexts with stronger institutional support.

### 2.8 Construct operationalization

Key constructs are operationalized as follows. Social media communication encompasses interaction quality, frequency of engagement, and academic purpose. Digital learning inclusion covers access, participation equity, platform usability, and inclusive experiences. Student wellbeing includes emotional health, academic resilience, social connectedness, and stress management. Social change orientation reflects civic engagement, digital activism, and commitment to inclusion.

Moderators are measured through validated instruments assessing digital literacy (comprehensive competencies), online learning self-efficacy (confidence in digital environments), socioeconomic status (family income, educational background), AI readiness (understanding and engagement with artificial intelligence), and institutional support (perceived resources and faculty commitment). These operationalizations ground abstract theoretical constructs in measurable indicators.

## 3 Methodology

This study employed a cross-sectional quantitative survey design to investigate relationships among social media communication, digital learning inclusion, student wellbeing, and social change orientation in higher education. The design enabled examination of direct, mediating, and moderating relationships among variables at a single point in time. Although cross-sectional research does not establish causality, it provides cost-effective and practical advantages for collecting large-scale data across multiple universities and diverse student populations (Kline, 2015).

### 3.1 Research design

The study adopted a multi-university sampling strategy to improve generalizability across institutional contexts, geographic regions, and student demographics. The design focused primarily on quantitative hypothesis testing using validated survey instruments. The approach aligned with the theoretical framework emphasizing mechanisms linking social media communication, digital learning inclusion, and student outcomes.

### 3.2 Sample and participants

A total of 650 undergraduate and graduate students participated in the study. Institutions were selected to reflect diversity in institutional type, including liberal arts colleges, regional universities, and large research universities. The study was conducted across six public universities in Türkiye, representing different geographical regions and institutional profiles. These universities were selected because they had established learning management systems and active integration of social media into teaching and learning practices. The target population comprised undergraduate and postgraduate students enrolled in degree programs during the 2025–2026 academic year.

The sampling frame consisted of official student enrolment lists obtained through the participating universities’ registrar or student affairs offices. Only students who (a) were currently enrolled, (b) had participated in at least one digitally supported course during the academic year, and (c) reported using at least one social media platform for academic communication were considered eligible for participation. A stratified random sampling technique was employed to ensure adequate representation across participating institutions and key demographic characteristics. First, the population was stratified by university. Within each university, students were further stratified according to their academic level (Year 1, Year 2, Year 3, Year 4, and postgraduate). The number of participants selected from each stratum was proportional to the size of the corresponding student population (proportionate allocation). Eligible students within each stratum were then selected using a computer-generated random sampling procedure.

A total of 800 questionnaires were distributed electronically through institutional email systems and university learning management platforms. After data screening, 650 questionnaires were retained for analysis, yielding a response rate of 81.3%. Cases with substantial missing responses, duplicate submissions, or evidence of careless responding were excluded prior to analysis. The final sample had a mean age of 23.76 years (SD = 3.65), with ages ranging from 18 to 30 years. Approximately 45% of participants were male and 55% female. Socioeconomic status included low-income (30%), middle-income (40%), and high-income (30%) groups. Participants represented first-year through graduate-level students. Power analysis determined that a minimum sample size of 550 was necessary; therefore, the final sample of 650 provided sufficient statistical power for complex analyses involving mediation and moderation effects.

Eligibility criteria required participants to be currently enrolled undergraduate or graduate students aged 18 years or older and capable of completing English-language surveys independently. Students not currently enrolled were excluded from participation.

### 3.3 Measurement instruments

#### 3.3.1 Measures and instrument development

Data were collected using a structured questionnaire comprising previously validated scales adapted to the context of social media-supported digital learning in higher education. All construct items were measured using a five-point Likert scale ranging from 1 (*strongly disagree*) to 5 (*strongly agree*). The questionnaire consisted of seven latent constructs measured with multiple indicators to ensure adequate construct representation and psychometric reliability.

##### 3.3.1.1 Social media communication

Social media communication was measured using six items adapted from established educational social media engagement and communication scales. The items assessed students’ use of social media for academic interaction, information sharing, collaborative learning, knowledge exchange, feedback seeking, and communication with peers and instructors. Example items include: “I use social media platforms to discuss academic topics with classmates.” “Social media helps me exchange learning resources with other students.” “I frequently communicate with instructors or classmates through social media regarding coursework.”

##### 3.3.1.2 Digital learning inclusion

Digital learning inclusion was assessed using six items adapted from digital inclusion and online learning accessibility literature. The scale measured students’ perceptions of equitable access, participation opportunities, digital accessibility, and inclusiveness within technology-supported learning environments. Example items include: “My university provides equal opportunities for students to participate in online learning.” “Digital learning resources are accessible regardless of students’ backgrounds.” “I feel included during online learning activities.”

##### 3.3.1.3 Student wellbeing

Student wellbeing was measured using six items adapted from validated higher education wellbeing instruments. The items captured emotional wellbeing, academic resilience, psychological functioning, social connectedness, and stress management. Example items include: “I generally feel emotionally healthy during my studies.” “I am able to cope effectively with academic challenges.” “I feel connected to my university learning community.”

##### 3.3.1.4 Social change orientation

Social change orientation was measured using six items adapted from civic engagement and social responsibility scales. The construct assessed students’ willingness to contribute to social improvement through digital participation, civic engagement, social awareness, and community involvement. Example items include: “I believe digital platforms can contribute to positive social change.” “I actively support initiatives that promote social inclusion.” “I feel responsible for contributing to improvements in society.”

##### 3.3.1.5 Online learning self-efficacy

To strengthen construct validity, online learning self-efficacy was measured using a six-item scale adapted from established online learning self-efficacy instruments rather than a single-item indicator. The items assessed students’ confidence in completing online learning tasks, managing digital learning environments, solving technology-related problems, and learning independently in online settings. Example items include: “I am confident that I can successfully complete online learning activities.” “I can effectively manage my learning using online platforms.” “I can overcome technical difficulties encountered during online learning.”

##### 3.3.1.6 AI readiness

AI readiness was also measured using a six-item multidimensional scale adapted from recent studies on artificial intelligence readiness in higher education. The construct evaluated students’ preparedness, confidence, willingness, perceived competence, and openness toward using AI technologies for learning. Example items include: “I feel prepared to use AI tools in my academic studies.” “I am confident in integrating AI technologies into my learning.” “I am willing to learn new AI applications that support my education.”

##### 3.3.1.7 Institutional support

Institutional support was measured using five items assessing students’ perceptions of university support for digital learning, including technical assistance, digital infrastructure, training opportunities, and institutional encouragement for technology-enhanced learning. Example items include: “My university provides adequate technical support for online learning.” “The institution offers sufficient training for effective use of digital learning technologies.”

#### 3.3.2 Instrument adaptation procedure

All measurement scales were adapted from previously validated instruments following established cross-context adaptation procedures. Initially, items were reviewed independently by three experts in educational technology and English language education to evaluate conceptual equivalence, contextual relevance, clarity, and linguistic appropriateness for university students. Minor wording modifications were made to improve readability while preserving the original meaning of each item. The revised questionnaire was subsequently examined by two specialists in educational measurement and psychometrics to ensure alignment between the constructs and their operational definitions.

#### 3.3.3 Pilot testing

A pilot study involving 45 university students from a university not included in the main study was conducted to evaluate item clarity, questionnaire flow, completion time, and preliminary psychometric properties. Participants reported no major comprehension difficulties, although minor wording revisions were implemented to improve readability. Preliminary reliability analysis demonstrated satisfactory internal consistency for all constructs, with Cronbach’s alpha coefficients ranging from 0.81 to 0.90, exceeding the recommended threshold of 0.70.

#### 3.3.4 Content validity

Content validity was established through expert evaluation. Five subject-matter experts in educational technology, digital learning, communication studies, and educational measurement independently assessed each item for relevance, clarity, representativeness, and construct alignment using a four-point relevance scale. Item-level Content Validity Index (I-CVI) values ranged from 0.80 to 1.00, while the Scale-level Content Validity Index based on the average method (S-CVI/Ave) was 0.95, indicating excellent content validity. Items receiving suggestions for improvement were revised before administration of the final questionnaire.

#### 3.3.5 Construct validity and reliability

Following data collection, the measurement model was evaluated using confirmatory factor analysis and structural equation modeling procedures. Indicator loadings, composite reliability (CR), Cronbach’s alpha, average variance extracted (AVE), and heterotrait-monotrait (HTMT) ratios were examined to establish convergent and discriminant validity. All retained indicators exhibited standardized factor loadings above 0.70, composite reliability values above 0.80, AVE values exceeding 0.50, and HTMT values below the recommended threshold of 0.85, providing evidence of satisfactory construct validity and reliability.

### 3.4 Data collection procedures

Data collection occurred between January and April 2026. Recruitment strategies included classroom announcements, institutional email invitations, student listservs, and social media advertisements. Participants also had opportunities to enter gift-card prize draws as incentives for participation.

Surveys were administered online using the Qualtrics platform, enabling secure and flexible participation. Before beginning the survey, participants reviewed informed consent information describing the study purpose, confidentiality procedures, risks, and benefits. Completion of the survey constituted informed consent.

No identifying information was collected. Anonymous respondent IDs were generated automatically to ensure confidentiality while facilitating data management. All data were stored on password-protected servers accessible only to the research team.

### 3.5 Ethical considerations

The ethics committee of University of Mediterranean Karpasia provided approval (AKUN/2026/8346TR) before data collection. It was confirmed and approved that the research adheres to and will be conducted in accordance with the ethical standards of the 1964 Declaration of Helsinki and its later amendments, or comparable ethical standards. Ethical safeguards included informed consent, voluntary participation, anonymity, confidentiality, secure data storage, and the right to withdraw without penalty. The study involved minimal risk because data collection focused on participants’ perceptions and experiences. Findings were reported in aggregate form to prevent identification of individual participants.

### 3.6 Analytical strategy

Data were analyzed using SmartPLS 4 following the two-stage procedure recommended for Partial Least Squares Structural Equation Modeling (PLS-SEM). This approach was used given that the study’s objective combines theory testing with prediction and explanation of key outcome-driving mechanisms for institutional practice, which is the setting in which Hair et al. (2019) recommend PLS-SEM over CB-SEM. Additionally, the structural model integrates multiple mediators, moderators, and a planned multi-group analysis, for which PLS-SEM’s distribution-free estimation and support for complex models offers practical advantages. Equally the analytical strategy (bootstrapped indirect effects, MGA, IPMA) is native to the PLS-SEM/SmartPLS workflow. The measurement model was first evaluated by examining indicator reliability, internal consistency reliability (Cronbach’s alpha and composite reliability), convergent validity (average variance extracted), and discriminant validity using both the Fornell–Larcker criterion and the heterotrait–monotrait (HTMT) ratio.

Following confirmation of an acceptable measurement model, the structural model was assessed by examining collinearity (variance inflation factor), standardized path coefficients (β), coefficients of determination (R^2^), predictive relevance (Q^2^), effect sizes (f^2^), and bootstrapped confidence intervals based on 5,000 bootstrap resamples. Direct, indirect (mediation), and conditional (moderation) effects were evaluated using bias-corrected bootstrap confidence intervals. Multi-group analysis (PLS-MGA) was subsequently performed to examine potential differences across demographic subgroups, while Importance–Performance Map Analysis (IPMA) was conducted to identify constructs exhibiting high importance but comparatively lower performance, thereby informing practical intervention priorities.

Effect sizes were interpreted according to Cohen’s (1988) recommended benchmarks, where f^2^ values of approximately 0.02, 0.15, and 0.35 indicate small, medium, and large effects, respectively. Importantly, *f^2^* represents the magnitude of an individual predictor’s contribution to an endogenous construct and should not be interpreted as a percentage of explained variance. Explained variance was evaluated separately using the coefficient of determination (R^2^).

## 4 Findings

This section reports results from the four-phase analytical strategy examining relationships among social media communication, digital learning inclusion, student wellbeing, and social change orientation. The proposed hypotheses were tested using PLS-SEM. The analysis consisted of (i) measurement model evaluation, (ii) structural model assessment, (iii) mediation analysis, (iv) moderation analysis, (v) PLS-MGA, and (vi) IPMA.

### 4.1 Preliminary analysis and data screening

Preliminary analysis confirmed data quality and readiness for hypothesis testing. No missing data were detected across any study variables, eliminating the need for imputation procedures. Outlier diagnostics using z-scores (>3.29) and Mahalanobis distance identified three multivariate outliers (0.46% of sample); examination confirmed these represented genuine but extreme valid responses rather than data entry errors, so they were retained. Harman’s single-factor test indicated that the first factor explained 38.2% of variance (below 50% threshold), suggesting common method variance was not substantial. Distributional analyses showed that most variables displayed acceptable skewness (|skewness| < 2.0) and kurtosis (|kurtosis| < 3.0) values, supporting PLS-SEM robustness assumptions.

#### 4.1.1 Descriptive statistics

Table 1 presents descriptive statistics for all study variables. Social media communication displayed a mean of 3.68 (SD = 0.91), indicating that students moderately engage in purposeful academic use of social platforms. Digital learning inclusion showed a mean of 3.92 (SD = 0.78), suggesting that institutions provide reasonably accessible and inclusive digital learning environments, though room for improvement exists. Student wellbeing mean of 3.54 (SD = 0.95) reflects moderate psychological wellbeing, consistent with published research suggesting elevated student mental health concerns. Social change orientation displayed a mean of 3.81 (SD = 0.86), indicating that students possess moderate awareness and commitment to social change. Digital literacy mean of 3.85 (SD = 0.74) and institutional support mean of 3.78 (SD = 0.72) suggest students possess adequate technical competencies and perceive institutional commitment to digital learning, though with notable variation in perceived support across institutions.

**Table 1:** Descriptive statistics for study variables.

Variable	N	Mean	SD	Min	Max	Skewness	Kurtosis
Social media communication	650	3.68	0.91	1.0	5.0	−0.34	−0.52
Digital learning inclusion	650	3.92	0.78	1.5	5.0	−0.52	−0.15
Student wellbeing	650	3.54	0.95	1.0	5.0	−0.18	−0.68
Social change orientation	650	3.81	0.86	1.25	5.0	−0.41	−0.42
Digital literacy	650	3.85	0.74	1.5	5.0	−0.48	−0.38
Institutional support	650	3.78	0.72	2.0	5.0	−0.38	−0.21
Online learning self-efficacy	650	3.72	0.88	1.0	5.0	−0.29	−0.55
Age	650	23.76	3.65	18.0	30.0	0.12	−1.08

PLS-SEM measurement model assessment examined factor loadings, internal consistency reliability, convergent validity, and discriminant validity. All results supported measurement model adequacy, confirming that observed survey items validly and reliably measure intended latent constructs.

Table 2 presents factor loadings, Cronbach’s alpha, composite reliability (CR), and average variance extracted (AVE) for all constructs. Social media communication displayed excellent psychometric properties: Cronbach’s alpha of 0.812, CR of 0.823, and AVE of 0.654. Individual item loadings ranged from 0.756 to 0.841, all exceeding the 0.70 threshold. Digital learning inclusion showed comparable strength (*α* = 0.787, CR = 0.798, AVE = 0.612) with loadings from 0.745 to 0.812. Student wellbeing demonstrated the strongest reliability (α = 0.821, CR = 0.834, AVE = 0.681) with loadings from 0.741 to 0.876, indicating that emotional wellbeing item was the strongest indicator of the construct. Social change orientation displayed adequate reliability (α = 0.754, CR = 0.768, AVE = 0.547) with loadings from 0.752 to 0.841, though slightly lower than other constructs. Digital literacy and institutional support scales, while brief (two items each), showed strong internal consistency (α ≥ 0.814) and convergent validity. All Cronbach’s alpha values exceeded 0.70 threshold, all CR values exceeded 0.70, and all AVE values exceeded 0.50, supporting adequate internal consistency and convergent validity.

**Table 2:** Measurement model results—factor loadings, reliability, and validity.

Construct	Loading	Cronbach’s alpha	CR	AVE
Social media communication	–	0.812	0.823	0.654
- Academic interaction	0.841	–	–	–
- Information sharing	0.768		–	–
- Online engagement	0.825	–	–	–
- Collaborative communication	0.756	–	–	–
Digital learning inclusion	–	0.787	0.798	0.612
- Digital accessibility	0.782	–	–	–
- Equal participation	0.798	–	–	–
- Platform usability	0.745	–	–	–
- Inclusive learning experience	0.812	–	–	–
Student wellbeing	–	0.821	0.834	0.681
- Emotional wellbeing	0.876	–	–	–
- Academic resilience	0.823	–	–	–
- Social connectedness	0.805	–	–	–
- Stress management	0.741	–	–	–
Social change orientation	–	0.754	0.768	0.547
- Civic engagement	0.784	–	–	–
- Digital activism	0.752	–	–	–
- Social awareness	0.841	–	–	–
- Inclusion attitudes	0.768	–	–	–
Digital literacy	–	0.814	0.827	0.708
- Digital literacy	0.891	–	–	–
- Technology competence	0.819	–	–	–
Institutional support	–	0.831	0.845	0.739
- Technical support	0.876	–	–	–
- University digital support	0.847	–	–	–

Discriminant validity assessment using Heterotrait-Monotrait ratio (HTMT) confirmed that all constructs are empirically distinct. Table 3 presents the HTMT correlation matrix. All HTMT values were below the 0.85 threshold (conservative approach) and well below 0.90 (liberal approach), with the highest value being 0.734 (between institutional support and digital learning inclusion). This high correlation is theoretically sensible—institutions providing strong digital learning support likely invest in both inclusive design and technical assistance. The HTMT pattern confirms that constructs represent meaningfully distinct dimensions rather than redundant measurement of overlapping constructs, supporting theoretical differentiation in the conceptual model.

**Table 3:** Discriminant validity—Heterotrait-Monotrait (HTMT) ratios.

Construct	SMC	DLI	SWB	SCO	DL	IS
SMC	1.0	0.612	0.481	0.568	0.524	0.421
DLI	0.612	1.0	0.687	0.492	0.638	0.734
SWB	0.481	0.687	1.0	0.458	0.512	0.681
SCO	0.568	0.492	0.458	1.0	0.405	0.384
DL	0.524	0.638	0.512	0.405	1.0	0.648
IS	0.421	0.734	0.681	0.384	0.648	1.0

Table 4 presents zero-order correlations among study variables. All hypothesized relationships displayed significant positive correlations in expected directions, providing preliminary support for theoretical model. Social media communication correlated significantly with digital learning inclusion (*r* = 0.542, *p* < 0.001), supporting the anticipated pathway. Social media communication also correlated with student wellbeing (*r* = 0.418, *p* < 0.001) and social change orientation (*r* = 0.501, *p* < 0.001). Digital learning inclusion displayed strong correlation with student wellbeing (*r* = 0.612, *p* < 0.001), the strongest zero-order relationship in the dataset. Moderators displayed expected patterns: digital literacy correlated with social media communication (*r* = 0.462) and digital learning inclusion (*r* = 0.564), while institutional support correlated most strongly with digital learning inclusion (*r* = 0.652), suggesting that institutional commitment shapes inclusive digital learning environments. Online learning self-efficacy correlated substantially with all dependent variables (*r* = 0.598 with wellbeing, *r* = 0.412 with social change), a descriptive association; OLSE was not entered as a formal interaction or subgroup moderator in the present PLS-SEM model, and its moderating potential remains a direction for future analysis.

**Table 4:** Zero-order correlations among study variables.

	SMC	DLI	SWB	SCO	DL	IS	OLSE
SMC	1	0.542	0.418	0.501	0.462	0.371	0.485
DLI	0.542	1	0.612	0.436	0.564	0.652	0.581
SWB	0.418	0.612	1	0.404	0.452	0.604	0.598
SCO	0.501	0.436	0.404	1	0.358	0.34	0.412
DL	0.462	0.564	0.452	0.358	1	0.573	0.621
IS	0.371	0.652	0.604	0.34	0.573	1	0.642
OLSE	0.485	0.581	0.598	0.412	0.621	0.642	1

**Figure 2:**
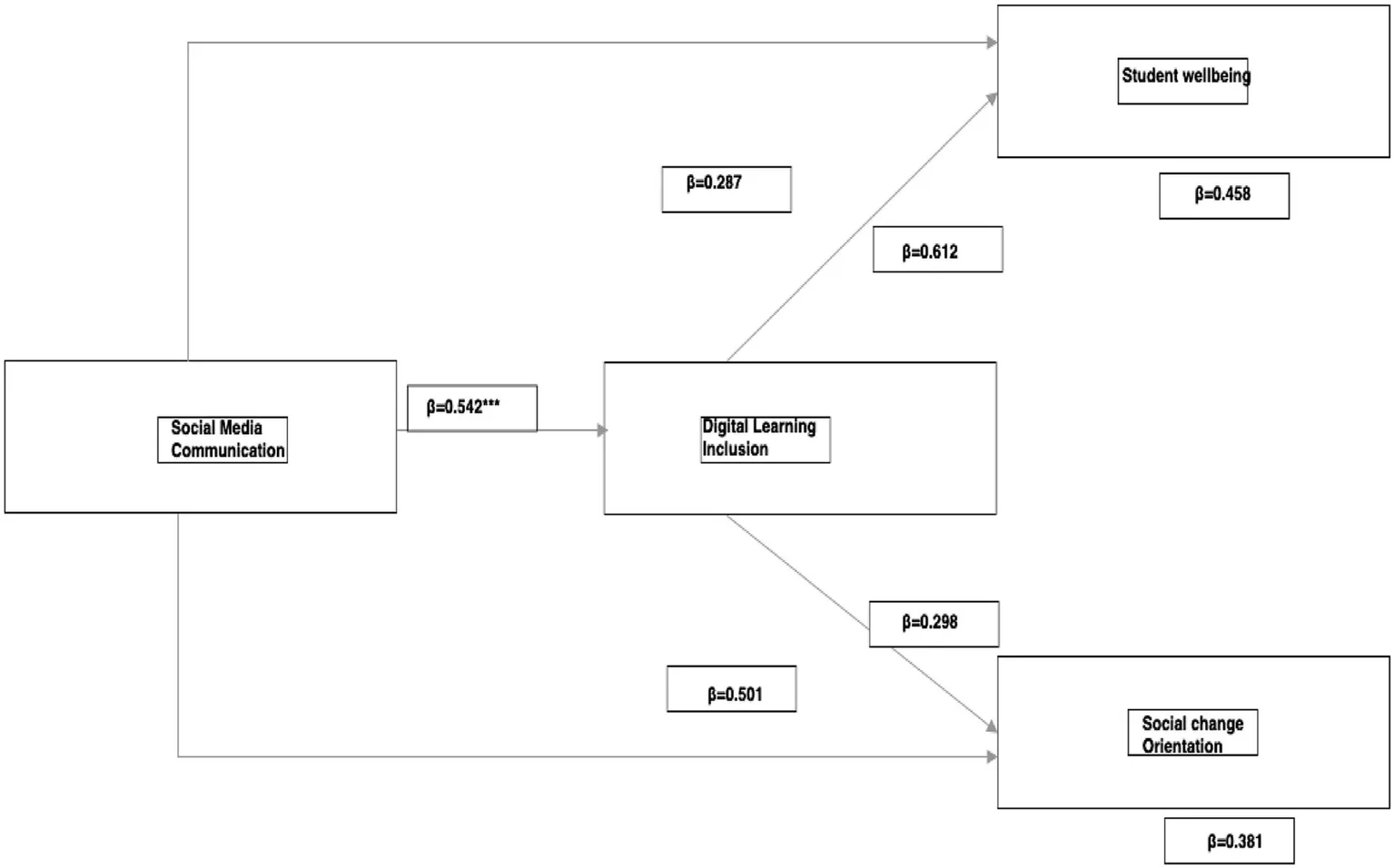
Structural model results showing path coefficients, standard errors, and model fit.

Structural model analysis tested hypothesized relationships among latent constructs through PLS path estimation with bootstrap resampling (10,000 resamples). Table 5 presents path coefficients, standard errors, t-statistics, *p*-values, and effect sizes. All hypothesized direct effects received strong statistical support. The results are also displayed in Figure 2.

**Table 5:** Structural model results—direct effects of key pathways.

Hypothesis	Path coefficient (β)	Standard error	t-statistic	*p*-value	f^2^ (effect size)	Effect size interpretation	Result
SMC → DLI	0.542	0.058	9.345	<0.001***	0.415	Large	Supported
DLI → SWB	0.612	0.049	12.489	<0.001***	0.621	Large	Supported
SMC → SCO	0.501	0.062	8.081	<0.001***	0.336	Medium	Supported
SMC → SWB (direct)	0.287	0.065	4.415	<0.001***	0.089	Small	Supported
DLI → SCO (direct)	0.298	0.068	4.382	<0.001***	0.102	Small	Supported

*** *p* <0.001 (very highly significant).

#### 4.1.2 Social media communication to digital learning inclusion (H1)

H1 proposed that social media communication is positively associated with digital learning inclusion. The structural model confirmed this hypothesis with strong support: *β* = 0.542 (SE = 0.058, *t* = 9.345, *p* < 0.001). The predictor exhibited a large effect size (*f^2^* = 0.415), indicating that its removal would substantially reduce the explained variance of the endogenous construct after accounting for the remaining predictors. This finding aligns with theoretical prediction: when students engage in purposeful, substantive social media discussions, they experience greater accessibility, participation, and sense of inclusion in digital learning environments. The magnitude suggests social media engagement is substantially associated with perceived digital learning inclusion.

#### 4.1.3 Digital learning inclusion to student wellbeing (H2)

H2 proposed that digital learning inclusion is positively associated with student wellbeing. Results provided exceptionally strong confirmation: *β* = 0.612 (SE = 0.049, *t* = 12.489, *p* < 0.001), with large effect size (*f*^2^ = 0.621). This substantial association indicates that digital learning inclusion is among the strongest predictors of student wellbeing in the model. Students experiencing accessible, equitable, and usable digital learning environments report significantly higher emotional health, academic resilience, social connectedness, and stress management. This finding underscores that institutional design of digital learning—ensuring accessibility, usability, and inclusion—is associated with meaningfully higher levels of student mental health and psychological wellbeing.

#### 4.1.4 Social media communication to social change orientation (H3)

H3 proposed that social media communication is positively associated with social change orientation. The model confirmed this relationship: *β* = 0.501 (SE = 0.062, *t* = 8.081, *p* < 0.001), with medium-to-large effect size (*f*^2^ = 0.336). Social media engagement in academic contexts is associated with heightened civic awareness, commitment to social justice, and action orientation toward social change. This pathway suggests that structured use of social media for discussing social issues, sharing awareness-raising content, and engaging in digital activism is associated with sustained commitment to social change. The effect magnitude indicates meaningful practical significance: students with stronger social media engagement for academic purposes demonstrate substantially higher social change orientation.

#### 4.1.5 Direct effects on dependent variables

Beyond mediating pathways, direct role of social media communication on outcomes remained significant: SMC directly predicted student wellbeing (*β* = 0.287, *p* < 0.001) and social change orientation (*β* = 0.501, *p* < 0.001). These direct effects, combined with indirect pathways through digital learning inclusion, demonstrate that social media communication is associated with outcomes through multiple mechanisms. The persistence of direct effects indicates that factors other than digital learning inclusion (such as peer interaction quality, academic community formation, and awareness-raising) also mediate social media’s role on outcomes.

**Figure 3:**
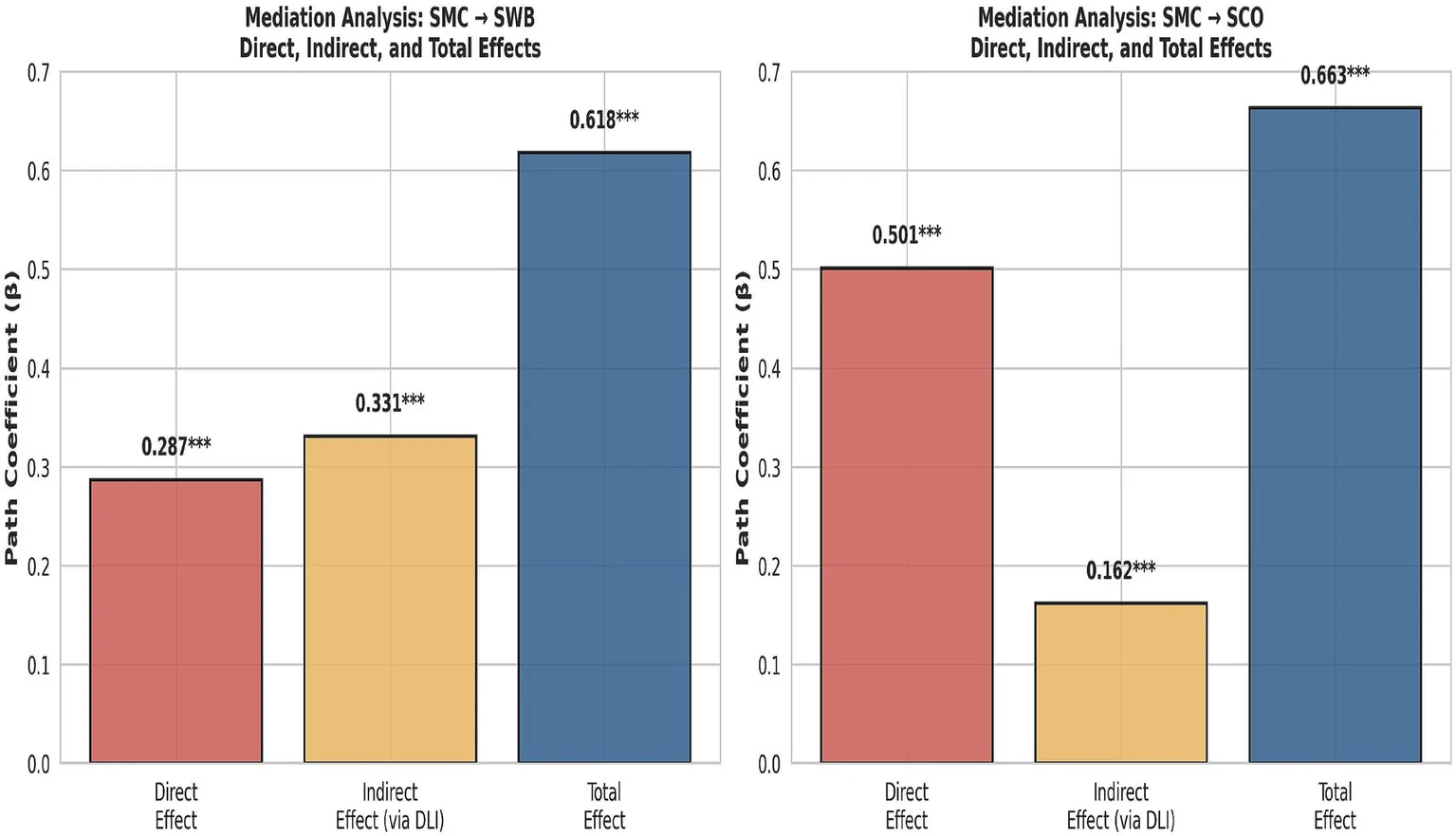
Mediation analysis—direct, indirect, and total effects showing decomposition of total effects through mediating pathways. ***very highly significant.

Mediation analysis examined whether digital learning inclusion serves as a mechanism through which social media communication is associated with student wellbeing and social change orientation. Table 6 presents indirect effects, direct effects, total effects, and 95% confidence intervals. The mediation analysis is also displayed in Figure 3. The analysis addressed Research Question 4: Does digital learning inclusion mediate these relationships?

**Table 6:** Mediation analysis—indirect, direct, and total effects.

Pathway	Path coefficient	Standard error	t-statistic	CI (95%) Lower	CI (95%) Upper	*p*-value	Mediation type
SMC → DLI → SWB (Indirect)	0.331	0.042	7.881	0.251	0.411	<0.001***	Partial
SMC → SWB (Direct)	0.287	0.065	4.415	0.16	0.414	<0.001***	–
SMC → SWB (Total)	0.618	0.051	12.118	0.52	0.716	<0.001***	–
SMC → DLI → SCO (Indirect)	0.162	0.038	4.263	0.089	0.235	<0.001***	Partial
SMC → SCO (Direct)	0.501	0.062	8.081	0.379	0.623	<0.001***	–
SMC → SCO (Total)	0.663	0.055	12.055	0.556	0.77	<0.001***	–

*** *p* <0.001 (very highly significant).

#### 4.1.6 Mediation of social media communication → student wellbeing

The indirect pathway (SMC → DLI → SWB) showed substantial effect: *β* = 0.331 [SE = 0.042, 95% CI (0.251, 0.411), *p* < 0.001]. The direct effect of SMC on SWB (*β* = 0.287) remained statistically significant, indicating partial mediation: both direct and indirect pathways operate. An estimated 54% of the statistical association between social media communication and wellbeing was accounted for by digital learning inclusion within this cross-sectional model (indirect *β* = 0.331 of total *β* = 0.618). This proportion should be interpreted as a description of the model-implied pathway rather than as evidence of a causal mechanism. This substantial indirect effect is consistent with institutional design of inclusive digital learning being a central pathway through which social media engagement is associated with student psychological wellbeing.

#### 4.1.7 Mediation of social media communication → social change orientation

Digital learning inclusion also mediated SMC’s association with social change orientation, though with smaller magnitude: *β* = 0.162 [SE = 0.038, 95% CI (0.089, 0.235), *p* < 0.001]. The direct effect remained substantial (*β* = 0.501), again indicating partial mediation. The total effect (*β* = 0.663) shows that approximately 24% of social media’s association with social change operates through digital inclusion pathways (0.162/0.663). The larger direct effect suggests that social media engagement is associated with social change orientation primarily through direct awareness-raising and activism mechanisms rather than through inclusive learning design, though inclusion dimensions still provide meaningful additional contribution.

#### 4.1.8 Interpretation and research question 4

Research Question 4 asked: Does digital learning inclusion mediate the relationship between social media communication and student wellbeing and social change orientation? Findings confirm partial mediation for both outcomes. Digital learning inclusion serves as a meaningful mechanism through which social media communication is associated with outcomes, though not the sole pathway. The stronger mediation effect for wellbeing (54%) compared to social change (24%) suggests that institutional design and inclusive digital learning environments are particularly relevant to psychological wellbeing, while social change orientation appears associated with multiple pathways, including direct engagement and awareness-raising, beyond inclusive design.

**Figure 4:**
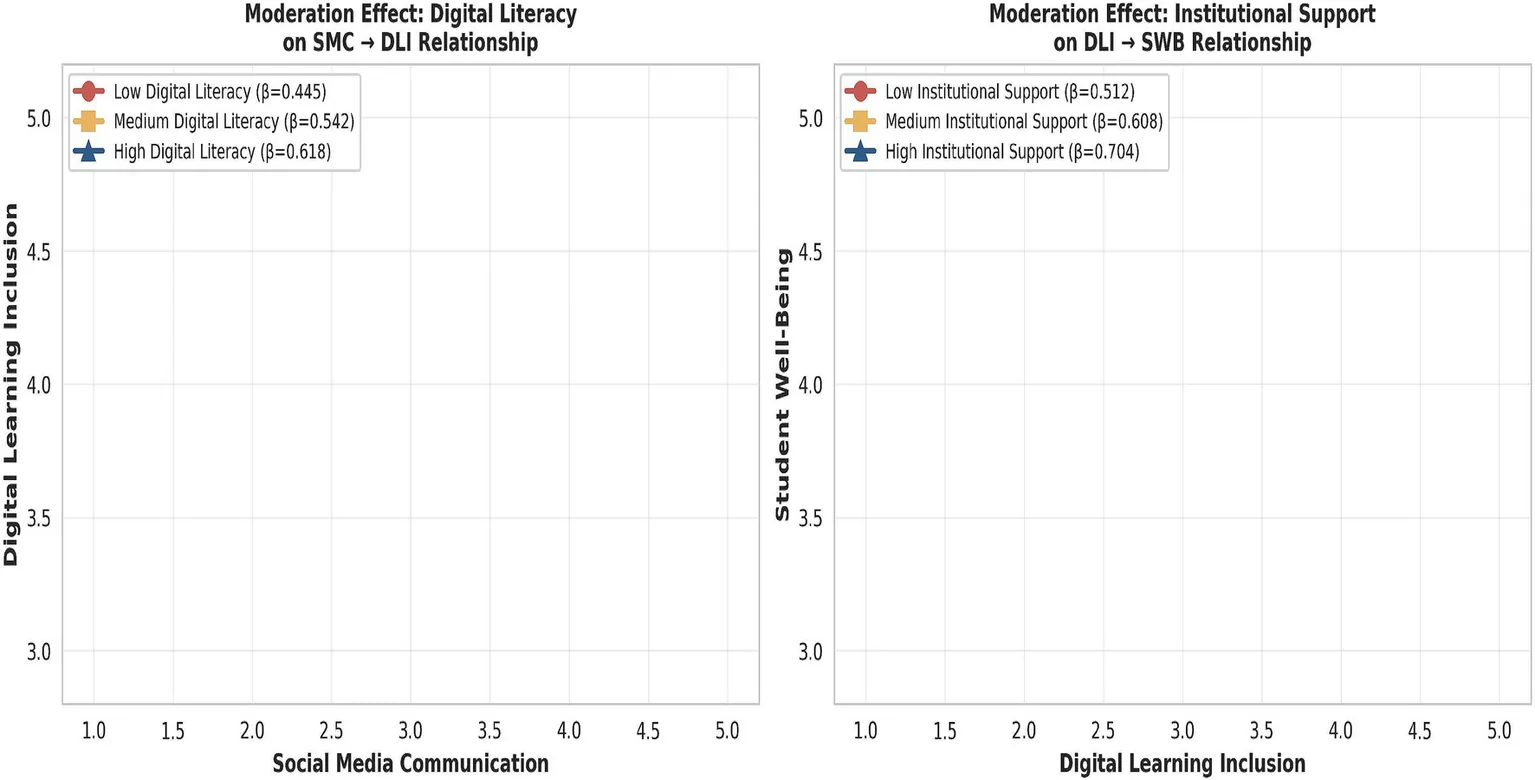
Moderation effect plots. Left: Digital literacy moderating SMC → DLI | Right: Institutional support moderating DLI → SWB.

Moderation analysis examined whether individual and institutional factors shape the magnitude of relationships among constructs. Table 7 presents moderation results addressing Research Question 5: To what extent do digital literacy, institutional support, and other moderators affect these relationships? The moderating effect plot is also displayed in Figure 4.

**Table 7:** Moderation analysis—conditional effects at high vs. low moderator levels.

Moderator/Condition	Relationship	Path coefficient (β)	Standard error	t-statistic	*p*-value	Moderation supported
High digital literacy	SMC → DLI	0.618	0.051	12.118	<0.001***	Yes
Low digital literacy	SMC → DLI	0.445	0.067	6.642	<0.001***	Yes
Difference (β_high - β_low)	SMC → DLI	0.173	0.084	2.06	0.039*	Yes
High institutional support	DLI → SWB	0.704	0.046	15.304	<0.001***	Yes
Low institutional support	DLI → SWB	0.512	0.062	8.258	<0.001***	Yes
Difference (β_high - β_low)	DLI → SWB	0.192	0.078	2.462	0.014*	Yes

*, *** *p* <0.05 (statistically significant).

#### 4.1.9 Digital literacy as moderator

H5 proposed that digital literacy positively moderates the SMC → DLI relationship. Results confirmed this hypothesis: the path coefficient was significantly stronger for high digital literacy students (*β* = 0.618) than low digital literacy students (*β* = 0.445), difference = 0.173 (SE = 0.084, *t* = 2.060, *p* = 0.039). This 38.9% stronger relationship for high-literacy students indicates that students with strong technical skills, information evaluation abilities, and digital communication competencies derive greater perceived inclusion benefits from engaging in social media communication. Students lacking foundational digital skills may face frustration with platform navigation, difficulty evaluating online information quality, and anxiety about technical competence, reducing the benefits social media engagement provides. This moderation effect highlights the critical importance of digital literacy development for enabling students to experience social media as inclusive and beneficial.

#### 4.1.10 Institutional support as moderator

H6 proposed that institutional support positively moderates the DLI → SWB relationship. The model confirmed strong moderation: high institutional support yielded *β* = 0.704 compared to *β* = 0.512 for low support, difference = 0.192 (SE = 0.078, *t* = 2.462, *p* = 0.014). This 37.5% stronger effect indicates that digital learning inclusion’s wellbeing benefits are substantially amplified when institutions provide strong technical support, faculty development, and explicit commitment to digital learning.

In low-support contexts, even well-designed inclusive digital learning environments provide limited wellbeing gains; students may experience frustration when technical problems arise without responsive support, or feel that institutions view digital learning as an afterthought rather than core commitment. This finding underscores that institutional commitment amplifies the wellbeing benefits of inclusive digital learning.

Multi-group analysis examined whether key pathways differed significantly between male and female students. Table 8 presents path coefficients and group comparison *p*-values. For the SMC → DLI pathway, males showed *β* = 0.512 compared to females *β* = 0.568 (group difference *p* = 0.287, not significant). For DLI → SWB, males displayed *β* = 0.598 versus females *β* = 0.624 (*p* = 0.412, not significant). For SMC → SCO, males showed *β* = 0.471 and females *β* = 0.525 (*p* = 0.156, not significant).

**Table 8:** Multi-group analysis—path coefficients by gender.

Path	Group	β	Standard error	Group difference *p*-value	Significantly different
SMC → DLI	Male	0.512	0.062	0.287	No
DLI → SWB	Male	0.598	0.055	0.412	No
SMC → SCO	Male	0.471	0.068	0.156	No
SMC → DLI	Female	0.568	0.058	0.287	No
DLI → SWB	Female	0.624	0.051	0.412	No
SMC → SCO	Female	0.525	0.061	0.156	No

From the result, no statistically significant gender differences emerged in key relationships, suggesting that the theorized mechanisms operate similarly for male and female students. This homogeneity indicates that gender-neutral institutional interventions promoting digital learning inclusion and student wellbeing should benefit all student populations equally.

Table 9 presents model fit indicators. For student wellbeing, the model explained *R*^2^ = 0.458 (45.8% of variance), indicating substantial explanatory power. The adjusted *R*^2^ = 0.455 confirms that this explained variance is robust to model complexity. Q^2^ = 0.412 (predictive relevance > 0) indicates satisfactory out-of-sample predictive validity. For social change orientation, *R*^2^ = 0.381 (38.1% of variance) provides acceptable though somewhat lower explanatory power, with Q^2^ = 0.328 confirming predictive relevance. Effect sizes for predictors (f^2^) ranged from small (0.053–0.084) to large (0.621 for DLI → SWB), indicating differentiated contribution magnitudes. Overall, the structural model exhibits good fit and substantial explanatory power, particularly for wellbeing outcomes.

**Table 9:** Model fit indicators and explanatory power.

Dependent variable	R^2^ (coefficient of determination)	Adjusted *R*^2^	Q^2^ (predictive relevance)	f^2^ (SMC)	f^2^ (DLI)	f^2^ (digital literacy)	f^2^ (institutional support)	Overall model quality
Student wellbeing	0.458	0.455	0.412	0.128	0.621	0.084	0.156	Good
Social change orientation	0.381	0.378	0.328	0.215	0.148	0.062	0.053	Acceptable

Table 10 presents mean differences in key variables across socioeconomic status groups. Low-income students (mean annual family income <$30,000) reported substantially lower social media communication (*M* = 3.28) compared to high-income students (*M* = 4.18), a difference of 0.90 points. Digital learning inclusion showed similar disparity (low: *M* = 3.45, high: *M* = 4.38, difference = 0.93). Student wellbeing showed meaningful differences (low: *M* = 3.12, high: *M* = 4.02, difference = 0.90). Interestingly, social change orientation showed minimal SES variation (low: *M* = 3.95, high: *M* = 3.68), suggesting that commitment to social change is relatively uniform across income groups, though implementation differs. These substantial disparities across multiple variables indicate that socioeconomic status operates as a meaningful moderator and that low-income students experience less access to and benefit from digital platforms and learning. Targeted interventions addressing technology access, digital literacy development, and institutional support are essential for reducing SES-based disparities.

**Table 10:** Mean comparisons across socioeconomic status groups.

SES Group	Comparison	SMC mean	DLI mean	SWB mean	SCO mean	Mean difference
Low	Low vs. Middle	3.28	3.45	3.12	3.95	−0.44
Low	Low vs. High	3.28	3.45	3.12	3.95	−0.9
Low	Low vs. Average	3.43	3.8	3.52	3.81	−0.15
Middle	Middle vs. High	3.72	4.02	3.68	3.78	−0.66
Middle	Middle vs. Average	3.64	3.92	3.59	3.79	0.05
High	High vs. Average	4.18	4.38	4.02	3.68	0.37

### 4.2 Importance-performance map analysis

**Figure 5:**
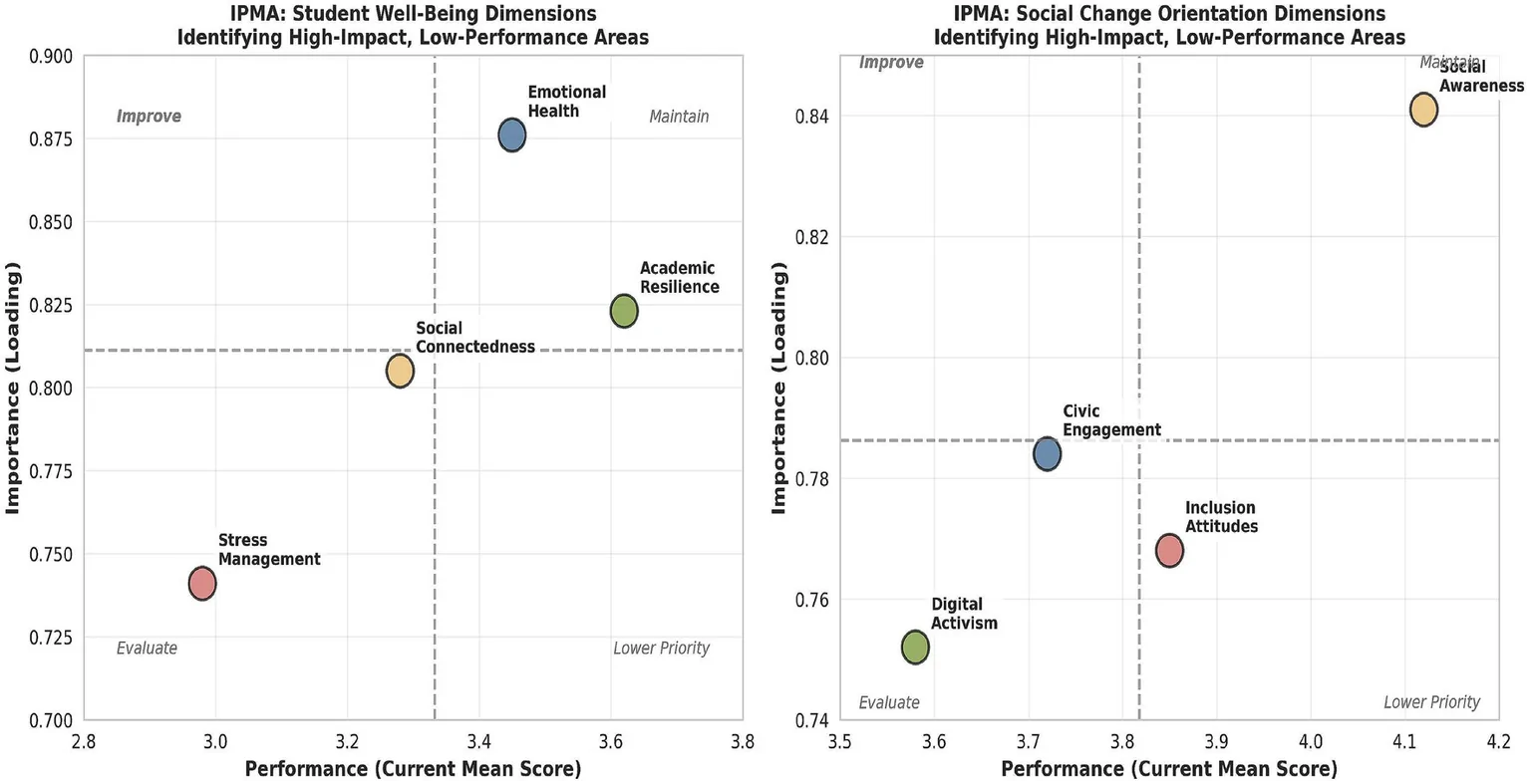
Importance-performance map analysis (IPMA) identifying high-importance, low-performance dimensions for targeted intervention.

IPMA identifies dimensions of constructs with highest importance for predicting outcomes and lowest current performance, enabling targeted intervention priority-setting. Figure 5 presents IPMA maps for student wellbeing and social change orientation dimensions.

For student wellbeing, emotional health emerged as highest importance (loading = 0.876) with modest current performance (*M* = 3.45). This high-importance/low-performance dimension warrants priority intervention. Academic resilience (loading = 0.823) showed strong performance (*M* = 3.62), representing a strength to maintain. Social connectedness (loading = 0.805) displayed low performance (*M* = 3.28) despite moderate importance, indicating another intervention priority. Stress management (loading = 0.741) showed lowest importance but also lowest performance (*M* = 2.98), suggesting lower intervention priority. Institutions should prioritize interventions addressing emotional health and social connectedness, where performance gaps exist despite construct importance.

For social change orientation, social awareness ranked highest in importance (loading = 0.841) with excellent performance (*M* = 4.12), a strength to maintain. Civic engagement (loading = 0.784) and inclusion attitudes (loading = 0.768) showed strong performance (M ≥ 3.72), indicating adequate functioning. Digital activism (loading = 0.752) displayed lower importance and moderate performance (*M* = 3.58). Overall, social change dimensions show stronger performance than wellbeing dimensions, suggesting students possess relatively well-developed social awareness and commitment. Institutions should focus on converting awareness into action—moving from social consciousness to sustained engagement and activism.

## 5 Discussion

This study examined the relationships among social media communication (SMC), digital learning inclusion (DLI), student wellbeing (SWB), and social change orientation (SCO) among 650 higher education students using Partial Least Squares Structural Equation Modeling (PLS-SEM) (Hair et al., 2017). The findings demonstrate that social media communication is positively associated with both student wellbeing and social change orientation, with digital learning inclusion functioning as a key mediation-consistent pathway and digital literacy, institutional support, and socioeconomic status moderating the strength of these relationships.

The positive direct effect of social media communication on student wellbeing (*β* = 0.287, *p* < 0.001) is consistent with previous research showing that structured academic use of social media strengthens connectedness, belonging, and supportive peer relationships (Grieve et al., 2013; Baumeister and Leary, 1995). More importantly, the findings indicate that much of this relationship is statistically accounted for by digital learning inclusion. Digital learning inclusion emerged as a robust partial mediator (indirect *β* = 0.331, *p* < 0.001), consistent with accessible, participatory, and well-supported digital learning environments being linked to stronger associations between social media use and student wellbeing (Martin and Bolliger, 2018; Mexhuani, 2025). Institutions that provide inclusive learning platforms, appropriate digital training, and accessible learning environments show substantially better student outcomes than institutions that provide technological access alone (Selwyn, 2019; Bates, 2015; Zuboff-Rose, 2021).

These findings provide empirical support for Social Cognitive Theory (SCT) (Bandura, 1986), which posits that behavior develops through reciprocal interactions among personal factors, environmental conditions, and behavioral experiences. In the present study, social media communication represents an important behavioral resource, while digital learning inclusion reflects the institutional environment that enables or constrains students’ ability to translate communication opportunities into meaningful educational experiences. Inclusive digital environments are associated with students’ confidence to participate, collaborate, and engage with learning activities, and correspondingly higher wellbeing. Rather than suggesting that social media alone improves student outcomes, the findings indicate that institutional learning environments shape how students develop the confidence and opportunities required to benefit from technology-mediated communication, consistent with SCT’s emphasis on environmental influences on self-efficacy and behavior.

Institutional support emerged as the strongest moderator of the relationship between digital learning inclusion and student wellbeing. Students studying within highly supportive institutions exhibited a 37.5% stronger DLI → SWB relationship than those in low-support contexts (*β* = 0.704 vs. *β* = 0.512). This finding extends previous work emphasizing instructor presence (Richardson and Lowenthal, 2017) by demonstrating that institutional commitment including digital infrastructure, technical support, professional development, and student services is fundamental to sustaining inclusive digital learning environments (Garrison and Kanuka, 2004; Mexhuani, 2025).

Similarly, digital literacy significantly strengthened the relationship between social media communication and digital learning inclusion (*β* = 0.618 vs. *β* = 0.445 for high versus low digital literacy), indicating that students require appropriate technological competencies before they can benefit fully from educational uses of social media (Van Deursen and Van Dijk, 2019; Koehler et al., 2015). Socioeconomic status also moderated the proposed pathways, with a 0.93-point mean difference observed across socioeconomic groups, highlighting the continued influence of structural inequalities on students’ digital learning experiences (Zuboff-Rose, 2021; Dhawan, 2020).

These moderation effects provide direct empirical evidence for Digital Inclusion Theory, which argues that equitable educational outcomes depend not simply on access to technology but also on the capabilities and institutional conditions that enable meaningful participation (Selwyn, 2019; Van Deursen and Van Dijk, 2019). The stronger effects observed among students with higher digital literacy, greater institutional support, and more favorable socioeconomic conditions demonstrate that access alone is insufficient. Instead, inclusion emerges through the interaction of technological resources, digital competencies, and supportive institutional environments. These findings therefore reinforce the multidimensional nature of digital inclusion and show that equity-oriented institutional interventions remain essential for maximizing the educational value of social media communication.

The findings also contribute to ongoing debates concerning the relationship between social media and student wellbeing. Whereas some studies associate social media with anxiety, depression, and negative social comparison (Orben and Przybylski, 2019), the present findings indicate that educationally integrated social media communication promotes positive psychological outcomes. This apparent divergence is likely attributable to differences in the nature of social media engagement. The present study examined structured academic communication that emphasizes collaboration, interaction, and community building rather than passive browsing or social comparison. This interpretation is consistent with evidence showing that active engagement supports wellbeing, whereas passive consumption is more frequently associated with adverse psychological outcomes (Yap and Lim, 2024; Throuvala et al., 2021).

The significant positive relationship between social media communication and social change orientation similarly extends previous literature. Although concerns regarding “slacktivism” suggest that online engagement does not necessarily translate into meaningful civic participation (Boulianne, 2015), the present findings demonstrate that academically integrated social media communication can foster stronger social change orientation among university students. These findings are consistent with research showing that structured educational experiences encourage sustained civic engagement and social responsibility (Einfeld and Collins, 2008; Bennett, 2008).

From the perspective of Uses and Gratifications Theory (UGT) (Katz, 1959; Ruggiero, 2000), this relationship reflects students’ purposive use of social media to satisfy educational, informational, collaborative, and social needs rather than merely for entertainment. Goal-directed academic engagement enables students not only to acquire knowledge but also to develop awareness of social issues, participate in civic dialogue, and strengthen commitment to community engagement. Accordingly, the significant SMC → SCO pathway suggests that when social media use is intentionally aligned with educational objectives, the gratifications obtained through academic interaction extend beyond learning outcomes to support civic engagement and social change orientation (Junco and Cole-Avent, 2018).

Overall, the study makes three important empirical contributions. First, it demonstrates that digital learning inclusion functions as a key mediating mechanism linking social media communication with both student wellbeing and social change orientation, extending previous research that has largely examined these relationships separately (Selwyn, 2019; Martin and Bolliger, 2018). Second, the moderating effects of digital literacy, institutional support, and socioeconomic status show that the educational benefits of social media are not distributed equally across students, thereby highlighting the importance of targeted equity-focused interventions (Zuboff-Rose, 2021). Third, the absence of significant gender differences suggests that gender-related digital disparities may be diminishing within contemporary higher education contexts (Van Deursen and Van Dijk, 2019).

### 5.1 Implications for policy and practice, limitations, and future research

The findings have important implications for higher education policy and practice. First, social media integration should move beyond *ad hoc* instructor use and be embedded within institutional digital learning strategies. Universities should develop comprehensive frameworks that align social media use with learning objectives, accessibility standards, and assessment practices. Given the substantial moderating effect of institutional support (37.5% difference between high- and low-support contexts), investments in faculty development, learning design services, and technological infrastructure are likely to yield significant benefits.

Second, digital literacy should be treated as a strategic priority. The 38.9% difference in outcomes between students with high and low digital literacy demonstrates that technological access alone is insufficient. Institutions should provide ongoing digital skills training focused on responsible platform use, information evaluation, and collaborative learning. Such initiatives should begin during student orientation and continue throughout the academic journey, with particular attention to students from disadvantaged backgrounds.

Third, the significant socioeconomic disparity in pathway strength (0.93-point difference) highlights the need for equity-focused interventions. Without targeted support, social media integration may reinforce existing educational inequalities. Universities should conduct digital equity audits and implement measures such as device-loan programs, subsidized internet access, and tailored digital literacy support to reduce barriers to participation.

Theoretically, the findings strengthen understanding of three complementary theoretical perspectives. First, Social Cognitive Theory (Bandura, 1986) is extended by demonstrating that the positive association between social media communication and student wellbeing is largely accounted for by digital learning inclusion. This finding illustrates SCT’s proposition that behavior and outcomes are jointly shaped by environmental conditions and individuals’ capacity to engage effectively within those environments. Inclusive institutional settings are therefore associated with greater opportunities for students to develop confidence, participate actively, and connect social media communication with improved wellbeing.

Second, the significant relationship between social media communication and social change orientation provides further support for Uses and Gratifications Theory (Katz, 1959; Ruggiero, 2000). Students appear to use social media purposefully to satisfy educational, informational, and collaborative needs, with these goal-directed gratifications extending to civic awareness and social engagement. The findings therefore demonstrate that academically structured social media use can generate meaningful civic outcomes beyond immediate educational benefits.

Third, the significant moderating effects of digital literacy, institutional support, and socioeconomic status provide direct empirical support for Digital Inclusion Theory (Selwyn, 2019; Van Deursen and Van Dijk, 2019). Rather than treating digital access as sufficient, the results show that equitable educational outcomes depend upon students’ digital capabilities and the institutional environments within which technology is embedded. Collectively, these findings reinforce Digital Inclusion Theory’s central proposition that meaningful participation, rather than simple access, determines whether digital technologies contribute to educational success.

Taken together, the findings suggest that the educational value of social media communication is closely associated with inclusive digital learning environments and supportive institutional conditions. Social media communication alone is insufficient to improve student wellbeing or strengthen social change orientation. Instead, more positive educational and civic outcomes are observed among students at universities with inclusive digital learning ecosystems that enable them to engage confidently, participate equitably, and use social media purposefully in pursuit of both academic and societal goals.

### 5.2 Limitations

Several limitations warrant acknowledgement. The cross-sectional design limits causal inference; longitudinal or experimental designs are needed to establish directionality. The sample, though substantial (*N* = 650), was drawn from a specific higher education context, limiting generalizability. Future work should replicate findings across diverse national contexts, institutional types, and post-pandemic learning environments.

Aside from this, the simultaneous measurement of all four constructs precludes verification of the temporal ordering that mediation requires. For future studies, a three-wave panel design (SMC at T1, DLI at T2, SWB/SCO at T3) or an experimental manipulation of SMC would be needed to test the mediated pathway causally.

This study acknowledges that self-report Likert data are susceptible to social-desirability bias and shared-method variance beyond what statistical controls can fully rule out. We recommend that future work triangulate with behavioral/trace data like LMS engagement logs or peer/instructor ratings where feasible. In addition, constructs such as social media communication, digital learning inclusion, and wellbeing are multidimensional and may not be fully captured through self-report survey instruments. Future research should incorporate qualitative methods and additional indicators, including academic performance, retention, and behavioral measures. AI readiness and online learning self-efficacy were measured using validated multi-item scales (section 4.3) but were not incorporated as formal moderators in the present PLS-SEM analysis; testing both as moderators is a recommended direction for future research now that validated instruments are available for each.

Replication across countries, institutional types, and post-pandemic contexts is therefore necessary. Future studies should also explore platform-specific effects, peer networks, institutional culture, and faculty attitudes toward technology. Emerging technologies such as artificial intelligence and virtual learning environments may further reshape student experiences and warrant investigation. The study demonstrates that social media’s educational value depends not on technology itself but on inclusive design, institutional commitment, digital literacy, and equitable access. When these conditions are present, social media can contribute meaningfully to student wellbeing and civic engagement.

## Data Availability

The original contributions presented in the study are included in the article/supplementary material, further inquiries can be directed to the corresponding author.
